# Long-term follow-up of insomnia patients: symptom trajectories and predictors of treatment outcomes in a mobile-based CBT-I program

**DOI:** 10.3389/fnins.2026.1753131

**Published:** 2026-03-18

**Authors:** Jiao Huang, Weijia Chen, Wentao Gao, Heng Zhao, Minghong Feng, Yumei Li, Fan Li, Ya Tan, Yuecheng Yang, Daijin Huang, Hong Shi

**Affiliations:** 1 Institute of Primate Translational Medicine, Kunming University of Science and Technology, Kunming, China; 2 Department of Neurosurgery, The First People's Hospital of Yunnan Province, The Affiliated Hospital of Kunming University of Science and Technology, Kunming, China; 3 Department of PET/CT Center, The First People's Hospital of Yunnan Province, The Affiliated Hospital of Kunming University of Science and Technology, Kunming, China; 4 Department of Endocrinology, Kunming Children's Hospital, Kunming, China; 5 Department of Pediatrics, Second Affiliated Hospital of Kunming Medical University, Kunming, China; 6 Department of Sleep Center, The First People's Hospital of Yunnan Province, The Affiliated Hospital of Kunming University of Science and Technology, Kunming, China

**Keywords:** anxiety, cognitive behavioral therapy (CBT), depression, insomnia, long-term follow-up, prognostic factors

## Abstract

**Objective:**

To investigate the longitudinal trajectories and prognostic factors of insomnia-related symptoms over a four-year follow-up period, using pharmacotherapy combined with a mobile-based cognitive behavioral therapy (CBT-I) for insomnia intervention.

**Methods:**

The clinical data including 1,022 patients of insomnia within January 2017 to January 2024 were obtained from the Sleep Center of the First People’s Hospital of Yunnan Province. Participants completed standardized assessments via the “Good Sleep 365” mobile app, including Pittsburgh Sleep Quality Index (PSQI), Patient Health Questionnaire-9 (PHQ-9), Generalized Anxiety Disorder-7 (GAD-7), PHQ-15, Epworth Sleepiness Scale (ESS), and Insomnia Severity Index (ISI) at baseline and multiple follow-up points. Symptom trends were analyzed descriptively, and linear mixed-effects models were used to examine trajectories and identify baseline predictors of treatment outcomes.

**Results:**

PSQI, GAD-7, PHQ-9, and PHQ-15 scores improved markedly during the first 12 months after treatment, but showed mild relapse thereafter, whereas ESS scores remained largely stable. Higher baseline PHQ-9 scores predicted poorer outcomes across all symptom domains, while baseline GAD-7 showed limited independent prognostic value after adjusting for depression. Older age was associated with better treatment response (*β* ≈ −0.05 for GAD7 and PHQ9).

**Conclusion:**

Baseline depressive symptoms were the strongest predictor of long-term outcomes across sleep, mood, and somatic domains, whereas anxiety added only modest prognostic value. These findings highlight the importance of routine depression screening to guide risk stratification and follow-up in chronic insomnia.

## 1 Introduction

Insomnia has become the second prevalent neuropsychiatric disorder globally, and it is associated with cardiovascular, metabolic, emotional, and neurodegenerative diseases (Neckelmann et al., 2007; Spiegelhalder et al., 2010; Wittchen et al., 2011; Xie et al., 2013; Zheng et al., 2019). Based World Health Organization (WHO) statistics, approximately 27% in the global populations suffer from sleep disorders (World Health Organization, 2017). The China Sleep Big Data Report (2023) revealed that the prevalence of insomnia in Chinese adults was 38.2%. Insomnia disproportionately affects women, the elderly, and those with mental health conditions (Ohayon, 2011). Longitudinal studies on insomnia in adults had reported the persistence rate from 20 to 69% (Morin et al., 2014), characterized insomnia a notable chronic course. Persistence rate is associated with age, 15% in children and adolescents, 42.7 and 28.2% in middle-aged women and men respectively (Zhang et al., 2012). Insomnia demonstrates natural remission and has a fluctuating clinical course, with remission rates ranging from 8 to 79% (Morin et al., 2009). The chronicity, variability and recurrence of insomnia contribute to differences in treatment response and complicate clinical management.

Classical longitudinal cohort work from the Zurich Study has further highlighted the heterogeneity of insomnia phenotypes and their psychiatric and somatic correlates. Vollrath et al. (1989) identified three subtypes of insomnia—occasional insomnia, repeated brief insomnia, and continued insomnia—and showed that these subtypes were differentially associated with recurrent brief depression, major depressive disorder, anxiety disorders, and a range of functional somatic syndromes. Importantly, more than half of individuals with insomnia were free of concurrent anxiety or depression at interview, and all three subtypes showed a similar tendency to relapse over a 7-year follow-up, indicating that insomnia can represent a partially independent and recurrent condition rather than merely a symptom of other psychiatric disorders (Vollrath et al., 1989). These findings underscore the need to consider both psychiatric comorbidity and longitudinal course when evaluating prognosis and tailoring interventions for insomnia.

Treatments for insomnia include pharmacotherapy, psychological and behavioral interventions (e.g., cognitive behavioral therapy, CBT-I) and other complementary and alternative therapies. CBT-I is the first-line treatment for achieving long-term efficacy (Espie et al., 2019; Perlis et al., 2022), but many factors could influence the treatment outcomes. Previous studies have demonstrated that multiple demographic and clinical factors were associated with the prognosis of CBT-I. In patients receiving mobile-based CBT-I, older age and delayed onset time have been identified as protective factors for favorable treatment prognosis, whereas lower educational level and higher anxiety severity (GAD-7 scores) were associated with poorer outcomes (Wei et al., 2024). In addition, male, lower educational level, and higher baseline Pittsburgh Sleep Quality Index (PSQI) scores have been reported as independent predictors of poorer CBT-I outcomes (Hu et al., 2022). In clinical, the bi-directional relationship between sleep disturbances and psychological distress further complicated management (Johnson et al., 2006). Poor sleep quality exacerbated anxiety and depression, in turn, elevated anxiety and depression levels could disrupt sleep patterns and hinder effective treatment (Roth, 2007).

The demographic and psychological characteristics could predict treatment outcomes in patients with insomnia. This study focuses on multi-year follow-up to investigate dynamic changes and prognostic factors in patients with insomnia. The multi-timepoint follow-up data over a four-year period were retrospectively analyzed to examine these trajectories in relation to PSQI, GAD-7, Patient Health Questionnaire-9(PHQ-9), PHQ-15, Epworth Sleepiness Scale (ESS), and Insomnia Severity Index (ISI). This study tries to clarify how baseline health indicators predict long-term symptom trajectories and to support personalized treatment strategies for chronic insomnia management.

## 2 Methods

### 2.1 Study subjects

This was a retrospective analysis of routinely collected app-based clinical data; no participants were prospectively recruited. The clinical data within January 2017 to January 2024 were obtained from the Sleep Center of the First People’s Hospital of Yunnan Province. Participants initiated mobile-based CBT-I on a rolling basis during this period; therefore, follow-up duration varied across individuals. The 48-month follow-up represents the maximum available follow-up prior to the database lock, and sample sizes at each follow-up time point were provided in Table 1. The data include gender, age, educational level and multiple scales. Scale assessments were conducted via the “Good Sleep 365” app, a mobile application designed for self-assessment among patients with insomnia. The “Good Sleep 365” app was primarily used in China as part of routine care in our sleep clinic. We were unable to locate a publicly accessible peer-reviewed randomized controlled trial (RCT) evaluating this specific app; however, the platform has been used in prior clinical research in real-world digital CBT-I settings (Liang et al., 2022). The app was provided by Hangzhou Sleep Health Clinic Co., Ltd., version 4.8.0.

**Table 1:** The baseline characteristics of patients at different follow-up points.

Variables	0 (*n* = 1,022)	0.5 (*n* = 522)	1 (*n* = 506)	2 (*n* = 337)	3 (*n* = 274)	6 (*n* = 283)	9 (*n* = 126)	12 (*n* = 225)	18 (*n* = 118)	24 (*n* = 116)	30 (*n* = 46)	36 (*n* = 55)	48 (*n* = 38)
Gender (*χ*^2^ = 17.147, *p* = 0.144)													
Male	310 (30.33%)	140 (26.82%)	124 (24.51%)	88 (26.11%)	74 (27.01%)	82 (28.98%)	33 (26.19%)	53 (23.56%)	39 (33.05%)	38 (32.76%)	16 (34.78%)	16 (29.09%)	16 (42.11%)
Female	711 (69.57%)	382 (73.18%)	382 (75.49%)	249 (73.89%)	200 (72.99%)	200 (70.67%)	93 (73.81%)	172 (76.44%)	79 (66.95%)	78 (67.24%)	30 (65.22%)	39 (70.91%)	22 (57.89%)
Age (Levene’s test *p* = 0.018^*^; Welch’s ANOVA *F* = 1.639, *p* = 0.077)													
	40.98 (13.20)	40.92 (12.93)	41.70 (12.91)	41.83 (12.54)	42.09 (12.62)	42.75 (13.10)	43.03 (12.45)	42.50 (12.26)	42.78 (11.92)	42.82 (10.19)	40.38 (11.01)	45.02 (11.80)	45.21 (11.61)
Education (*χ*^2^ = 34.57, *p* = 0.958)													
Primary school	109 (10.67%)	54 (10.34%)	62 (12.25%)	31 (9.20%)	24 (8.76%)	27 (9.54%)	14 (11.11%)	21 (9.33%)	13 (11.02%)	6 (5.17%)	1 (2.17%)	2 (3.64%)	1 (2.63%)
Middle school	376 (36.79%)	191 (36.59%)	182 (35.97%)	124 (36.80%)	111 (40.51%)	100 (35.34%)	51 (40.48%)	81 (36.00%)	45 (38.14%)	45 (38.79%)	14 (30.43%)	17 (30.91%)	11 (28.95%)
Undergraduate	488 (47.75%)	252 (48.28%)	244 (48.22%)	164 (48.66%)	123 (44.89%)	137 (48.41%)	57 (45.24%)	110 (48.89%)	54 (45.76%)	59 (50.86%)	27 (58.70%)	34 (61.82%)	24 (63.16%)
Master	40 (3.91%)	21 (4.02%)	16 (3.16%)	17 (5.04%)	15 (5.47%)	15 (5.30%)	4 (3.17%)	11 (4.89%)	4 (3.39%)	5 (4.31%)	3 (6.52%)	1 (1.82%)	2 (5.26%)
PHD	3 (0.29%)	2 (0.38%)	1 (0.20%)	0 (0.00%)	0 (0.00%)	1 (0.35%)	0 (0.00%)	0 (0.00%)	0 (0.00%)	0 (0.00%)	0 (0.00%)	0 (0.00%)	0 (0.00%)
BMI (Levene’s test *p* = 0.531; ANOVA *F* = 0.415, *p* = 0.9271)													
	21.45 (4.40)	21.58 (3.54)	21.39 (3.76)	21.64 (3.07)	21.42 (3.59)	21.10 (4.13)	21.19 (5.10)	21.06 (4.76)	21.27 (5.05)	22.07 (4.01)	21.53 (5.46)	22.14 (2.46)	21.76 (2.62)

Data are presented as mean (SD) or number (%). For categorical variables, chi-squares test were used. For continuous variables, if Levene’s Variance test *p*-value > 0.05, ANOVA was used; if *p*-value < 0.05, Welch’s ANOVA was applied. *p < 0.05.

The inclusion criteria were: (1) clinical diagnosis of insomnia meeting ICD-10 diagnostic criteria; (2) ability to use the “Good Sleep 365” app; (3) a follow-up duration of at least 0.5 months, with primary data derived from scale assessments. The exclusion criteria were: (1) secondary or comorbid insomnia resulting from psychiatric disorders; (2) insomnia is caused by psychoactive substances or physical illnesses. In the available database, patients who attended only a baseline visit without any post-baseline assessment were not captured; therefore, the analytic cohort necessarily comprised individuals with at least one follow-up assessment (≥0.5 months).

### 2.2 Study design

Patients within the inclusion criteria completed assessments via the app. Upon login, each patient was automatically assigned a unique identification code and required to provide basic demographic information, including gender, age, height, weight, and educational level. Subsequently, patients completed a set of standardized self-report questionnaires embedded in the app, including PSQI (Buysse et al., 1989), PHQ-9, GAD-7 (Spitzer et al., 2006), PHQ-15 (Kroenke et al., 2002), ESS (Johns, 1991) and ISI (Bastien et al., 2001). During each follow-up visit, patients re-logged into their existing accounts and to complete the same self-assessment scales. All evaluation data were submitted through the app. Follow-up assessments were collected as part of routine clinical return visits. At each visit, patients were required to complete the full set of in-app questionnaires and report current medication information via their existing account. All submissions were time-stamped and linked using the unique in-app user ID. Therefore, missing follow-up data largely reflected patients who did not return for subsequent visits.

All participants received mobile-based CBT-I in combination with routine pharmacotherapy. The CBT-I program was delivered within the application and was adapted from evidence-based CBT-I manuals (Qaseem et al., 2016; Riemann et al., 2017) and Chinese clinical guidelines for insomnia. The intervention consisted of the following core modules: (1) sleep restriction, (2) stimulus control, (3) cognitive restructuring, (4) sleep hygiene education, and (5) relaxation training. These modules were presented as brief video or audio sessions, each lasting approximately 1–4 min, accompanied by a daily electronic sleep diary. One new session was released each day, and patients were instructed to complete at least one session per week over a total of 8 weeks. The system automatically recorded log-ins, diary completion, and module completion in real time. While users were allowed to review previously completed content at any time, progression to new modules was strictly sequential and was unlocked based on completion of the preceding sessions; manual skipping of modules was not permitted.

Sleep restriction was introduced from week 1 in our app-based program and implemented in an algorithm-guided manner, consistent with standard CBT-I sleep restriction principles (Perlis et al., 2005)A fixed wake-up time was set, and the initial sleep window (time in bed) was prescribed based on the participant’s average total sleep time derived from the electronic sleep diary (typically the preceding week). The sleep window was constrained by a minimum of 5 h to reduce excessive daytime sleepiness. Bedtime was calculated by subtracting the prescribed sleep window from the fixed wake-up time. The sleep window was reviewed and adjusted weekly according to diary-derived sleep efficiency (sleep efficiency = total sleep time / time in bed × 100%): if sleep efficiency was >85%, the sleep window was increased by 15 min; if sleep efficiency was 80–85%, it was maintained; and if sleep efficiency was <80%, it was decreased by 15 min (without going below the minimum sleep window).

Routine pharmacotherapy was prescribed according to clinical judgment. Medication class and baseline dose were recorded descriptively; participants were required to be on a stable regimen prior to CBT-I, and those who initiated new medication at CBT-I start were excluded. Follow-up medication changes were not systematically recorded and were therefore not modeled.

### 2.3 Scale assessments

The PSQI was used to assess sleep quality over the past month. The global score ranges from 0 to 21, with higher scores indicating poorer sleep quality. A score between 6 and 10 indicates mild sleep disturbance, 11–15 indicates moderate sleep disturbance, and ≥16 indicates severe sleep disturbance (Xie et al., 2025). It consists of 7 components: (1) subjective Sleep Quality (2) time to fall asleep; (3) sleep time; (4) sleep efficiency (5) addition problem, such as nighttime awakenings, breathing difficulties, and nightmares; (6) drug; (7) day function. The GAD-7 is used to assess anxiety severity. A score≤4 indicates no anxiety, 5–9 indicates mild anxiety, 10–14 indicates moderate anxiety and a score ≥15 indicates severe anxiety (Spitzer et al., 2006). The PHQ-9 is a widely used tool for assessing depressive symptoms. A score≤4 indicates no depression, 5–9 indicates mild depression, 10–14 indicates moderate depression and a score ≥15 indicates severe depression (Kroenke et al., 2001). The ESS is used to assess daytime sleepiness. A score≤10 indicates normal range of daytime sleepiness, ≥11 points indicates excessive daytime sleepiness (Johns, 1991). The PHQ-15 is used to assess the severity of somatic symptom burden. A score≤4 indicates no somatic symptoms, 5–9 indicates mild somatic symptoms, 10–14 indicates moderate somatic symptoms and a score ≥15 indicates severe somatic symptoms (Kroenke et al., 2002). The ISI is a self-reported measure used to assess the subjective severity of insomnia. A score≤7 indicates no clinically significant insomnia, 8–14 indicates mild insomnia, 15–21 indicates moderate insomnia and a score ≥22 indicates severe insomnia (Bastien et al., 2001).

### 2.4 Statistical analysis

All statistical analyses were conducted in R version 4.4.2 (R Foundation for Statistical Computing, Vienna, Austria). Longitudinal data were analyzed using linear mixed-effects models (LMMs) implemented in the *lme4* and *lmerTest* packages. Longitudinal outcomes were defined as post-baseline questionnaire scores (≥0.5 months). Baseline (T0) scores were included only as covariates. Each model included a random intercept for participant ID to account for within-subject correlations over the follow-up period (up to 48 months). The dependent variables were repeated symptom scores, including the PSQI, ISI, GAD-7, PHQ-9, ESS and PHQ-15. As this was a retrospective, naturalistic observational study based on routinely collected app-based clinical data, no *a priori* sample size calculation was performed; the sample size was determined by the number of eligible participants available in the database during the study period (*N* = 1,022).

Follow-up month was entered as a fixed effect to model symptom trajectories over time. Other fixed effects included age, sex, education level, and baseline psychological and sleep-related measures (baseline GAD-7, PHQ-9, ESS, PHQ-15 and PSQI components). Models were fitted using restricted maximum likelihood (REML) estimation.

Missing data in covariates were handled using multiple imputation by chained equations (MICE) with predictive mean matching (PMM) for continuous variables (m = 5, maxit = 10), under a Missing at Random (MAR) assumption. Importantly, repeated outcome measures were not imputed; LMMs were fitted using all available outcome observations. Variables with low or moderate missingness (<20%) were imputed. Because BMI had a high missing rate (>50%), it was excluded from the primary models and examined only in exploratory sensitivity analyses. Model estimates were pooled across imputations using Rubin’s rules.

To evaluate robustness, sensitivity analyses were performed using: (1) joint models including both baseline GAD-7 and PHQ-9, (2) models including only one of them, and (3) residualized (“unique”) models based on the unique variance of each to address shared variance between anxiety and depression. In addition, to assess the potential impact of attrition on estimated time trends in this real-world dataset, we conducted an intention-to-treat sensitivity analysis using last observation carried forward (ITT/LOCF) on a prespecified discrete follow-up grid (0.5, 1, 2, 3, 6, 12, 24, 36, and 48 months) including baseline; participants who discontinued follow-up retained their last observed outcome value at subsequent time points. This ITT/LOCF analysis was intended as a sensitivity analysis to bound the potential influence of dropout-related missingness on estimated time trends, rather than as a definitive primary estimate.

Multicollinearity among predictors was assessed using generalized variance inflation factors (GVIFs) from the car package. All GVIF^(1/(2 × Df)) values were <2.0, indicating no problematic collinearity. Effect estimates were reported as *β* coefficients with 95% confidence intervals (CIs) and *p*-values. A two-tailed *α* = 0.01 was applied for the mixed-effects regression models. For each LMM, marginal (fixed effects) and conditional (fixed + random effects) *R*^2^ values were computed using Nakagawa’s method (performance package) and averaged across the imputed datasets. All statistical visualizations were produced using ggplot2 and broom.mixed, and forest plots were used to display fixed effects and their 95% CIs.

## 3 Results

### 3.1 General characteristics

A total of 1,022 patients were included in this study, 69.57% were female. The average age was 40.98 ± 13.20. Among them, 48.4% were 31–50 years old, and 22.5% were 18–30 years old. The mean body mass index (BMI) was 21.45 ± 4.40. A total of 47.75% held a bachelor’s degree, 36.79% had a middle school education, 10.67% had primary school education or below, and 4.2% had a postgraduate degree (Table 1).

PSQI scores indicated that 36.01% of participants had moderate sleep disturbance and 45.59% had severe sleep disturbance. ISI scores showed that over 58.09% were classified as having moderate to severe insomnia. According to GAD-7, 32.09% reported mild anxiety, 19.28% moderate anxiety, and 16.83% severe anxiety. PHQ-9 results showed that 31.12% had mild depression, 19.96% moderate depression, and 23.39% severe depression. Based on PHQ-15, 37.28% had mild somatic symptoms, 34.59% moderate, and 15.75% severe symptoms. ESS scores indicated that 85.15% of participants did not exhibit excessive daytime sleepiness (Table 2).

**Table 2:** Baseline distribution of symptom severity by scale scores.

Category	*n*	Proportion (%)
PSQI score (*n* = 1,022)		
0–5: Good sleep quality	46	4.5%
6–10: Mild sleep disturbance	148	14.48%
11–15: Moderate sleep disturbance	368	36.01%
16–21: Severe sleep disturbance	460	45.59%
GAD-7 score (*n* = 1,022)		
0–4: No anxiety	325	31.8%
5–9: Mild anxiety	328	32.09%
10–14: Moderate anxiety	197	19.28%
15–21: Severe anxiety	172	16.83%
PHQ-9 score (*n* = 1,022)		
0–4: No depression	261	25.54%
5–9: Mild depression	318	31.12%
10–14: Moderate depression	204	19.96%
≥15: Severe depression	239	23.39%
ESS score (*n* = 1,010)		
0–10: Normal daytime sleepiness	860	85.15%
>10: Excessive daytime sleepiness	150	14.85%
ISI score (*n* = 816)		
0–7: No insomnia	129	15.81%
8–14: Mild insomnia	213	26.1%
15–21: Moderate insomnia	314	38.48%
22–28: Severe insomnia	160	19.61%
PHQ-15 score (*n* = 1,003)		
0–4: No somatic symptoms	124	12.36%
5–9: Mild somatic symptoms	374	37.28%
10–14: Moderate somatic symptoms	347	34.59%
≥15: Severe somatic symptoms	158	15.75%

PSQI, Pittsburgh Sleep Quality Index; GAD-7, Generalized Anxiety Disorder-7; PHQ-9, Patient Health Questionnaire-9; ESS, Epworth Sleepiness Scale; ISI, Insomnia Severity Index; PHQ-15, Patient Health Questionnaire-15.

Among the 1,022 participants, 356 (34.87%) received benzodiazepines, 470 (45.99%) received non-benzodiazepine hypnotics, and 152 (14.89%) received melatonin receptor agonists. Antidepressants and/or anxiolytics were prescribed to participants who showed elevated anxiety or depression on assessment. Some patients received more than one class of medication concurrently. All participants were on a stable dose prior to starting CBT-I. These descriptive statistics provide an overview of routine pharmacotherapy administered alongside mobile-based CBT-I in this cohort.

### 3.2 Demographic distribution across different follow-up months

**Figure 1:**
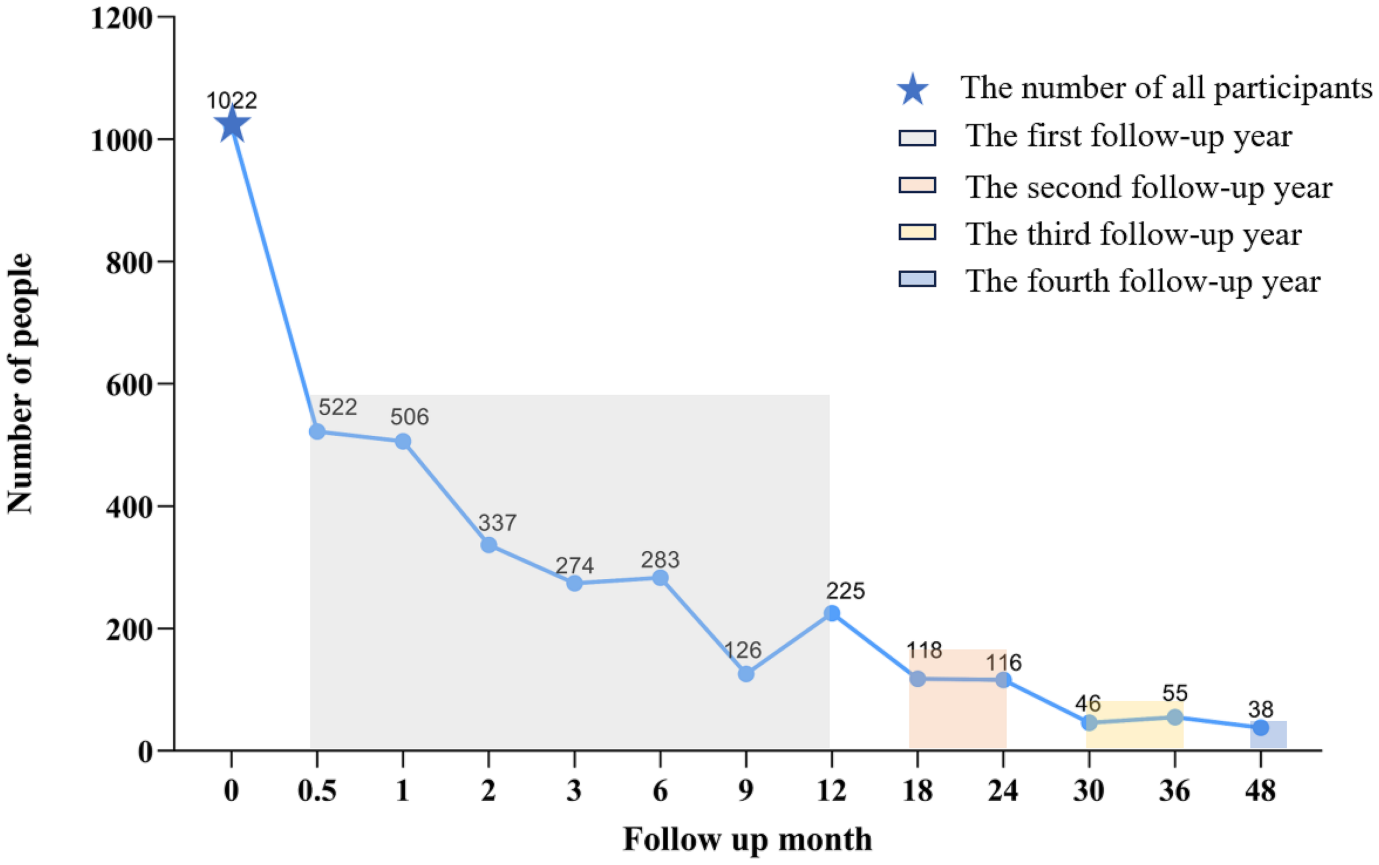
The distribution of participants across each follow-up month. The distribution of participants across each follow-up month showed a gradual decline in sample size over time.

The distribution of participants across each follow-up month showed a gradual decline in sample size over time (Figure 1). The demographic characteristics of patients at different follow-up points (0 to 48 months) were presented (Table 1). The data show that females consistently comprised most of the sample, with their proportion decreasing from 76.44% at month 12 to 57.89% at month 48. The gender distribution over time was not statistically significant (*p* = 0.144). The mean age of participants increased from 40.98 ± 13.20 years at baseline to 45.21 ± 11.61 years at month 48, with no statistically significant difference (*p* = 0.077). The distribution of educational level and BMI remained stable throughout the follow-up period (*p* = 0.9271 and *p* = 0.958, respectively) (Table 1).

### 3.3 Trends of different indicators over follow-up months

**Figure 2:**
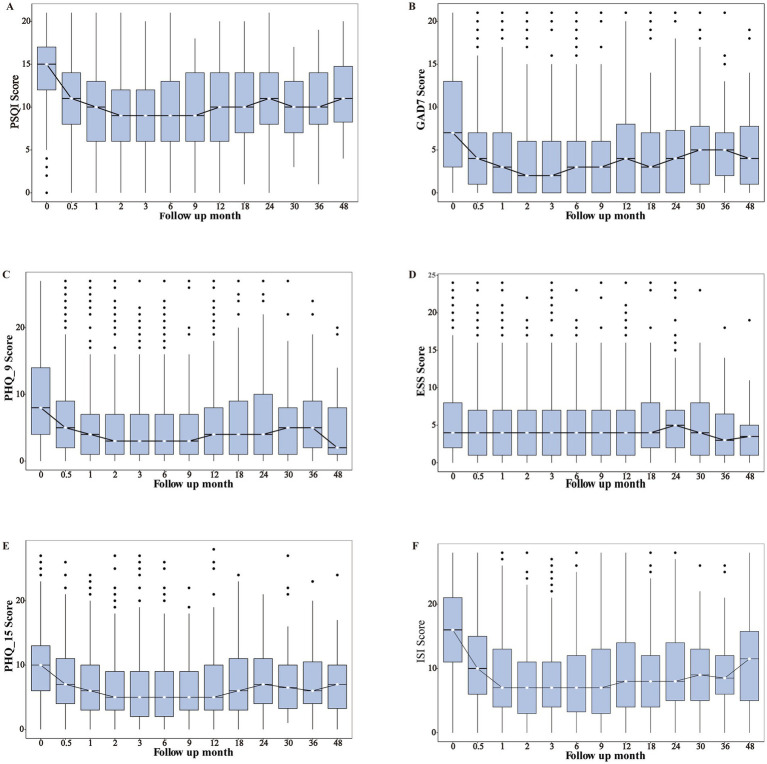
The trends of various scale assessments across the follow-up months. PSQI, Pittsburgh Sleep Quality Index; GAD-7, Generalized Anxiety Disorder-7; PHQ-9, Patient Health Questionnaire-9; ESS, Epworth Sleepiness Scale; ISI, Insomnia Severity Index; PHQ-15, Patient Health Questionnaire-15. For non-normally distributed continuous variables, the Kruskal–Wallis *H* test was used for group comparisons.

Improvements in PSQI, GAD-7, PHQ-9, and PHQ-15 scores were observed within 2 weeks post-treatment, became significant between month 1 and 9, and began to show fluctuations after month 12. ESS scores showed no significant changes over follow-up months (Table 3; Figure 2). The post-treatment trajectories of PSQI components exhibited heterogeneous patterns (Supplementary Figure 1). C1 (subjective sleep quality) was improved significantly within 0.5 months, with fluctuations emerging around month 12. C2 (sleep latency) was improved by month 1, remained stable until month 9, then fluctuated. C3 (sleep duration) was sustained improved with minimal variability. C4 (sleep efficiency) was improved by month 1, remained stable until month 30, then slightly rebounded. C5 (sleep disturbances) showed no notable change. C6 (sleep medication use) was stable in month 3, declined from months 6–18, rose after month 24, declined post-month 30, and was nearly absent by month 48. C7 (daytime dysfunction) initially worsened post-treatment, showed gradual improvement from month 1, and then stabilized.

**Table 3:** The trends of various scale assessments across the follow-up months.

Scale/Assessment	0 (*n* = 1,022)	0.5 (*n* = 522)	1 (*n* = 506)	2 (*n* = 337)	3 (*n* = 274)	6 (*n* = 283)	9 (*n* = 126)	12 (*n* = 225)	18 (*n* = 118)	24 (*n* = 116)	30 (*n* = 46)	36 (*n* = 55)	48 (*n* = 38)	*p*-value
PSQI	15 (12–17)	11 (7–14)	10 (6–13)	9 (6–12)	9 (6–12)	9 (6–13)	9 (6–14)	10 (6–14)	10 (7–14)	11 (8–14)	10 (7–13)	10 (8–14)	11 (8–14)	0****
GAD7	7 (3–13)	4 (1–7)	3 (0–7)	2 (0–6)	2 (0–6)	3 (0–6)	3 (0–6)	4 (0–8)	3 (0–7)	4 (0–8)	5 (1–8)	5 (2–7)	4 (1–7)	0****
PHQ9	8 (5–14)	5 (2–9)	4 (1–7)	3 (1–7)	3 (1–7)	3 (1–7)	3 (1–7)	4 (1–8)	4 (1–9)	4 (1–10)	5 (1–8)	5 (2–9)	2 (1–8)	0****
ESS	4 (2–8)	4 (1–7)	4 (1–7)	4 (1–7)	4 (1–7)	4 (1–7)	4 (1–7)	4 (1–7)	5 (2–8)	5 (2–7)	4 (1–8)	3 (1–6)	3 (1–5)	0.25
PHQ15	10 (6–13)	8 (5–11)	6 (3–10)	5 (3–9)	5 (2–9)	5 (2–9)	5 (3–9)	5 (3–10)	7 (3–11)	7 (4–11)	7 (4–10)	6 (4–10)	7 (3–10)	0****
ISI	16 (11–21)	10 (6–15)	7 (4–13)	7 (3–11)	7 (4–11)	7 (3–12)	7 (3–13)	8 (4–14)	8 (4–12)	8 (5–14)	9 (5–13)	8 (6–12)	11 (5–15)	0****
PSQI-C1	3 (2–3)	1 (1–2)	1 (0–2)	1 (0–2)	1 (0–2)	1 (0–2)	1 (0–2)	1 (1–2)	2 (1–2)	2 (1–2)	1 (1–2)	2 (1–2)	2 (1–2)	0****
PSQI-C2	3 (2–3)	1 (1–2)	1 (1–2)	1 (1–2)	1 (1–2)	1 (1–2)	1 (1–2)	2 (1–3)	2 (1–2)	2 (1–3)	2 (1–2)	2 (1–2)	1 (1–3)	0****
PSQI-C3	3 (2–3)	2 (1–3)	2 (0–3)	2 (0–2)	2 (0–2)	2 (0–3)	2 (0–3)	2 (0–3)	2 (0–3)	2 (1–3)	2 (0–2)	2 (0–3)	2 (2–3)	0****
PSQI-C4	3 (1–3)	1 (0–2)	1 (0–2)	1 (0–2)	1 (0–2)	1 (0–2)	1 (0–3)	1 (0–2)	1 (0–2)	1 (0–2)	1 (0–2)	2 (0–2)	2 (0–3)	0****
PSQI-C5	1 (1–2)	1 (1–1)	1 (1–1)	1 (1–1)	1 (1–1)	1 (1–1)	1 (1–1)	1 (1–1)	1 (1–1)	1 (1–1)	1 (1–1)	1 (1–1)	1 (1–1)	0****
PSQI-C6	1 (0–3)	3 (0–3)	3 (0–3)	3 (0–3)	3 (0–3)	2 (0–3)	1 (0–3)	1 (0–3)	2 (0–3)	3 (0–3)	1 (0–3)	0 (0–3)	0 (0–3)	0****
PSQI-C7	2(1–3)	3(2–3)	2 (1–3)	2 (1–3)	2 (1–3)	2 (1–3)	2 (1–2)	2 (1–2)	2 (1–2)	2 (1–3)	2 (1–3)	2 (1–3)	2 (1–3)	0****

Data were presented as median (interquartile range).

PSQI, Pittsburgh Sleep Quality Index; GAD-7, Generalized Anxiety Disorder-7; PHQ-9, Patient Health Questionnaire-9; ESS, Epworth Sleepiness Scale; ISI, Insomnia Severity Index; PHQ-15, Patient Health Questionnaire-15; C1, Subjective sleep quality; C2, Time to fall asleep; C3, Sleep time; C4, Sleep efficiency; C5, Addition problem; C6, Drug; C7, day function.

For non-normally distributed continuous variables, the Kruskal–Wallis H test was used for group comparisons. ****p < 0.001.

### 3.4 Linear mixed-effects regression analyses of symptom trajectories

Five longitudinal linear mixed-effects models were fitted with PSQI, GAD-7, PHQ-9, ESS, and PHQ-15 as dependent variables. All models included a random intercept for participant ID. In the main joint models including both baseline GAD-7 and PHQ-9, fixed effects explained approximately 9–30% of the variance in symptom scores (marginal *R*^2^ ≈ 0.09 for PSQI and 0.23–0.30 for the other outcomes), whereas fixed plus random effects together explained about 53–66% of the total variance (conditional *R*^2^ ≈ 0.53–0.66).

In the main joint models, follow-up month showed small positive slopes for PSQI (*β* = 0.021, *p* = 0.011), GAD-7 (*β* = 0.037, *p* < 0.001), and PHQ-9 (*β* = 0.022, *p* = 0.021), indicating a slight tendency toward worsening over time in observed follow-up data. At the prespecified *α* = 0.01 level, the time effect remained statistically significant for GAD-7, whereas the time effects for PSQI and PHQ-9 did not reach this threshold. No clear time effects were detected for ESS or PHQ-15.

Baseline symptom scores were the strongest predictors of their own follow-up trajectories. Higher baseline ESS, PHQ-15, GAD-7, and PHQ-9 were each strongly associated with higher repeated scores on the corresponding scales. Beyond these within-scale effects, baseline depressive symptoms (PHQ-9) predicted worse outcomes across multiple domains, including poorer sleep quality (PSQI: *β* = 0.091, *p* = 0.008), higher daytime sleepiness (ESS: *β* = 0.154, *p* < 0.001), and more somatic symptoms (PHQ-15: *β* = 0.122, *p* < 0.001).

In the joint models, baseline anxiety (GAD-7) was the main independent predictor of GAD-7 trajectories, but its associations with PHQ-9, ESS, and PHQ-15 were weak and non-significant after adjusting for baseline PHQ-9. In contrast, in GAD-only sensitivity models that excluded PHQ-9, baseline GAD-7 significantly predicted higher subsequent PHQ-9, ESS, and PHQ-15 scores, indicating that these cross-domain associations were largely attenuated once shared variance with depression was controlled.

Regarding demographics, older age was associated with lower GAD-7 and PHQ-9 scores over time. Female sex was associated with higher PHQ-15 scores, whereas effects on other outcomes were small and inconsistent. Education showed no robust associations.

Sensitivity analyses using PHQ-only and residualized “unique” models produced effect estimates and *R*^2^ values that were highly similar to those from the main joint models, supporting robustness to collinearity between GAD-7 and PHQ-9. Multicollinearity diagnostics indicated stable model specification, with all GVIF^(1/(2 × Df)) < 2.0 and the highest values observed for the GAD-7/PHQ-9 pair (≈1.6–1.8). Exploratory models including BMI in the first imputed dataset showed that BMI was not a significant predictor of any outcome (all *p* > 0.10) and did not materially change other fixed effects; given >50% missingness, BMI was therefore not retained in the primary models.

To address potential bias due to attrition in this real-world dataset, we additionally conducted an intention-to-treat sensitivity analysis using last observation carried forward (ITT/LOCF) on a prespecified discrete follow-up grid including baseline. In these ITT/LOCF models, follow-up month was consistently associated with decreases (improvements) in PSQI, GAD-7, PHQ-9, ESS, and PHQ-15 over time (all *p* < 0.001), indicating that the estimated longitudinal trajectories were sensitive to how missing follow-up data are handled.

**Figure 3:**
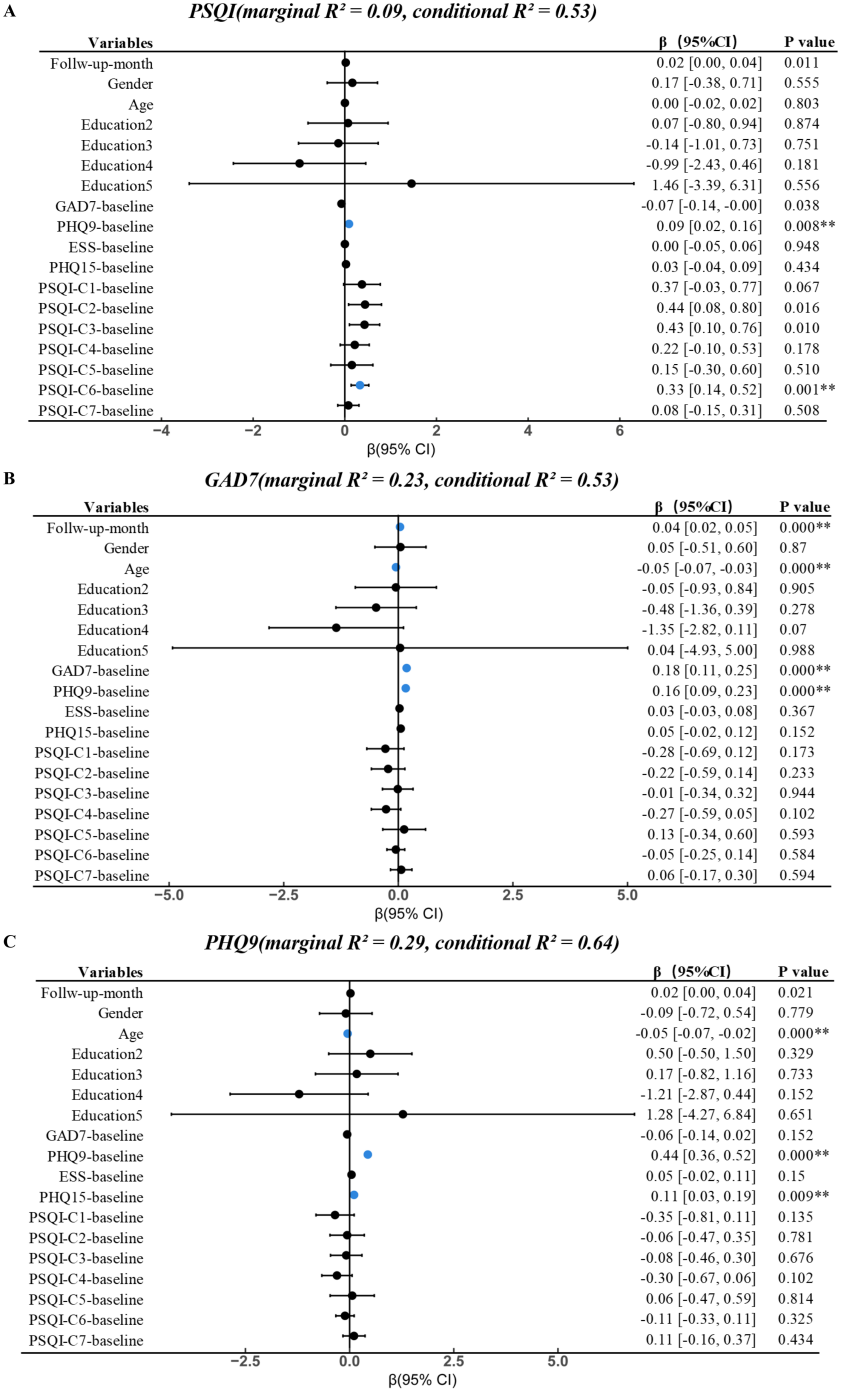
Linear mixed-effects models analysis of factors associated with prognostic. PSQI, Pittsburgh Sleep Quality Index; GAD-7, Generalized Anxiety Disorder-7; PHQ-9, Patient Health Questionnaire-9; ESS, Epworth Sleepiness Scale; PHQ-15, Patient Health Questionnaire-15; **p < 0.01 in blue.

All fixed-effect estimates with 95% confidence intervals for the main joint models were presented in Figure 3 and Supplementary Figure 2, with corresponding sensitivity analyses in Supplementary Table 1.

## 4 Discussion

Insomnia is a chronic condition with a fluctuating course, shaped by life events, psychological factors, and overall health status (Morin et al., 2009). CBT-I can significantly improve sleep quality, with treatment effects keep at least 6 months (Mitchell et al., 2019). In this study, a high remission rate was observed during treatment. Combined pharmacotherapy and mobile-based CBT-I provided a trajectory resembling the natural course of insomnia, and symptom recurrence is common occurrence.

### 4.1 Baseline depression as a central predictor of long-term outcomes

Across all mixed-effects models, baseline depressive symptoms (PHQ-9) emerged as a robust and consistent predictor of poorer prognosis. Higher PHQ-9 scores were associated with higher subsequent PSQI, GAD-7, PHQ-9, ESS, and PHQ-15 scores, indicating worse sleep quality, more severe emotional symptoms, greater daytime sleepiness, and heavier somatic burden over time. This broad pattern suggests that baseline depression captures a general liability to persistent symptom burden in patients with insomnia, extending beyond mood itself to sleep, daytime functioning, and physical complaints. These findings are consistent with longitudinal work showing that depression is strongly intertwined with the onset, maintenance, and recurrence of insomnia (Wei et al., 2024), and highlight the importance of routinely screening for and treating depressive symptoms in this population.

Clinically, the results implied that patients with high baseline PHQ-9 scores represented a high-risk subgroup that might require closer monitoring, shorter follow-up intervals, and earlier intensification or augmentation of treatment. Integrating targeted depression management—such as structured psychotherapy or optimized antidepressant strategies—into insomnia care might be essential for achieving sustained improvements. Conversely, patients with low baseline depression might benefit adequately from standard CBT-I with less intensive follow-up, which had implications for resource allocation in routine practice.

### 4.2 The role of baseline anxiety in the context of depression

In contrast to depression, baseline anxiety (GAD-7) showed a more restricted and unstable pattern of associations. GAD-7 scores strongly predicted the trajectory of GAD-7 itself but had only modest or statistically weak relationships with sleep and other outcomes once baseline PHQ-9 was included in the models. In anxiety-only sensitivity models that excluded PHQ-9, baseline GAD-7 predicted several outcomes more strongly, whereas in depression-only models PHQ-9 remained a consistent cross-domain predictor. Residualized “unique” scores and collinearity diagnostics further indicated that a substantial portion of the variance in GAD-7 overlapped with PHQ-9.

These findings suggested that the baseline anxiety was largely shared with and partly subsumed by depression. This did not imply that anxiety was unimportant, but rather that depression might represent the more parsimonious and clinically informative marker. Given the heterogeneity of anxiety and their overlap with depressive affect, future studies with finer-grained assessment of anxiety subtypes and severity are needed to clarify the specific contribution of anxiety to long-term outcomes in insomnia.

### 4.3 Age as a positive predictor of follow-up outcomes

Age was positively associated with follow-up outcomes, consistent with some prior findings (Wei et al., 2024). As an active treatment approach, mobile-based CBT-I requires patients to improve poor sleep hygiene and establish regular routines. Older individuals often retired or with reduced work demands, might be easier to follow treatment recommendations. A stable daily rhythm supported the development of a healthy sleep–wake cycle, facilitating improvements in daytime sleepiness and related somatic symptoms (Morin et al., 1999; McPhillips et al., 2024). However, evidence on digital/online CBT-I in older adults is mixed. Age-related comorbidities and reduced physical or social activity may complicate insomnia management, and barriers such as digital literacy and usability can reduce engagement with internet-based interventions. Therefore, the observed positive association of age in our naturalistic cohort may reflect selection and context (e.g., clinic-based follow-up and structured app use) rather than a universally higher efficacy of online CBT-I in older adults (Hughes, 2024; Ritterband et al., 2025).

PSQI was selected as the primary sleep outcome because it captures multidimensional sleep quality, which aligns with our aim to model long-term trajectories. ISI provides an overall index of insomnia severity; however, in this study it showed substantial overlap with PSQI (and PSQI components) and added limited incremental information, increasing redundancy/collinearity. Therefore, PSQI was prioritized as the primary modeled sleep outcome. As a limitation, PSQI may be influenced by comorbid sleep disorders, which were not systematically assessed in this dataset.

This naturalistic observational study cannot establish causality. All patients received routine pharmacotherapy based on symptom severity; medication type and dose were recorded descriptively but not modeled as separate predictors, so the trajectories reported here reflect the combined effects of CBT-I and usual care, and residual confounding by medication cannot be excluded. Medication changes during follow-up were not systematically captured and may have contributed to residual confounding.

Attrition over the 4-year follow-up was substantial, raising the possibility of selective dropout despite broadly similar baseline demographics among participants contributing data at different follow-up durations. Primary analyses used linear mixed-effects models fit to all available post-baseline outcome measurements; missingness in covariates was handled via multiple imputation under a Missing at Random (MAR) assumption. However, outcome missingness driven by non-attendance may be related to unmeasured factors (i.e., potentially informative dropout), and patients with baseline-only visits were not captured in the available database. Long-term time trends should therefore be interpreted cautiously and as exploratory, particularly beyond 12 months. Notably, estimated time effects differed by missing-data assumption: observed-data LMMs suggested flat or slightly worsening trajectories for some outcomes, whereas the prespecified ITT/LOCF analysis on a discrete follow-up grid (using baseline as the initial value and carrying forward the last observation after dropout) showed sustained improvement. This divergence indicates that conclusions about time trends are sensitive to assumptions about missing outcomes. In contrast, substantive findings for baseline predictors were robust across modeling strategies. Baseline symptom burden—particularly baseline depressive symptoms (PHQ-9)—remained a consistent cross-domain predictor of poorer outcomes, and baseline anxiety (GAD-7) primarily predicted its own trajectory with attenuated cross-domain associations after adjustment for PHQ-9; this pattern was reproduced in the joint, single-predictor, and residualized (“unique”) sensitivity models. Overall, attrition primarily affected the estimated time trends, while associations between baseline predictors and subsequent symptom levels were stable across analyses.

## 5 Conclusion

This study highlighted baseline depression as a central prognostic marker in patients with insomnia receiving combined pharmacotherapy and mobile-based CBT-I. Depressive symptoms were associated with a broad pattern of persistent difficulties across sleep, mood, daytime functioning, and somatic burden, whereas baseline anxiety contributed more modestly and largely through shared variance with depression. From a clinical perspective, incorporating baseline depression screening into routine assessment may help stratify follow-up intensity: patients with high PHQ-9 scores, high somatic symptom burden, and possibly women could be prioritized for closer monitoring and early augmentation of treatment, while those with lower depression levels and more stable life contexts might be managed with less intensive but structured digital follow-up. Such stratified strategies may support more personalized and efficient long-term management of chronic insomnia.

## Data Availability

The original contributions presented in the study are included in the article/Supplementary material, further inquiries can be directed to the corresponding authors.
