# Supplementary figures and images for: Long-term follow-up of insomnia patients: symptom trajectories and predictors of treatment outcomes in a mobile-based CBT-I program
Source: Front Neurosci. 2026 Mar 18;20:1753131. doi: 10.3389/fnins.2026.1753131 (PMC13038904; doi:10.3389/fnins.2026.1753131)

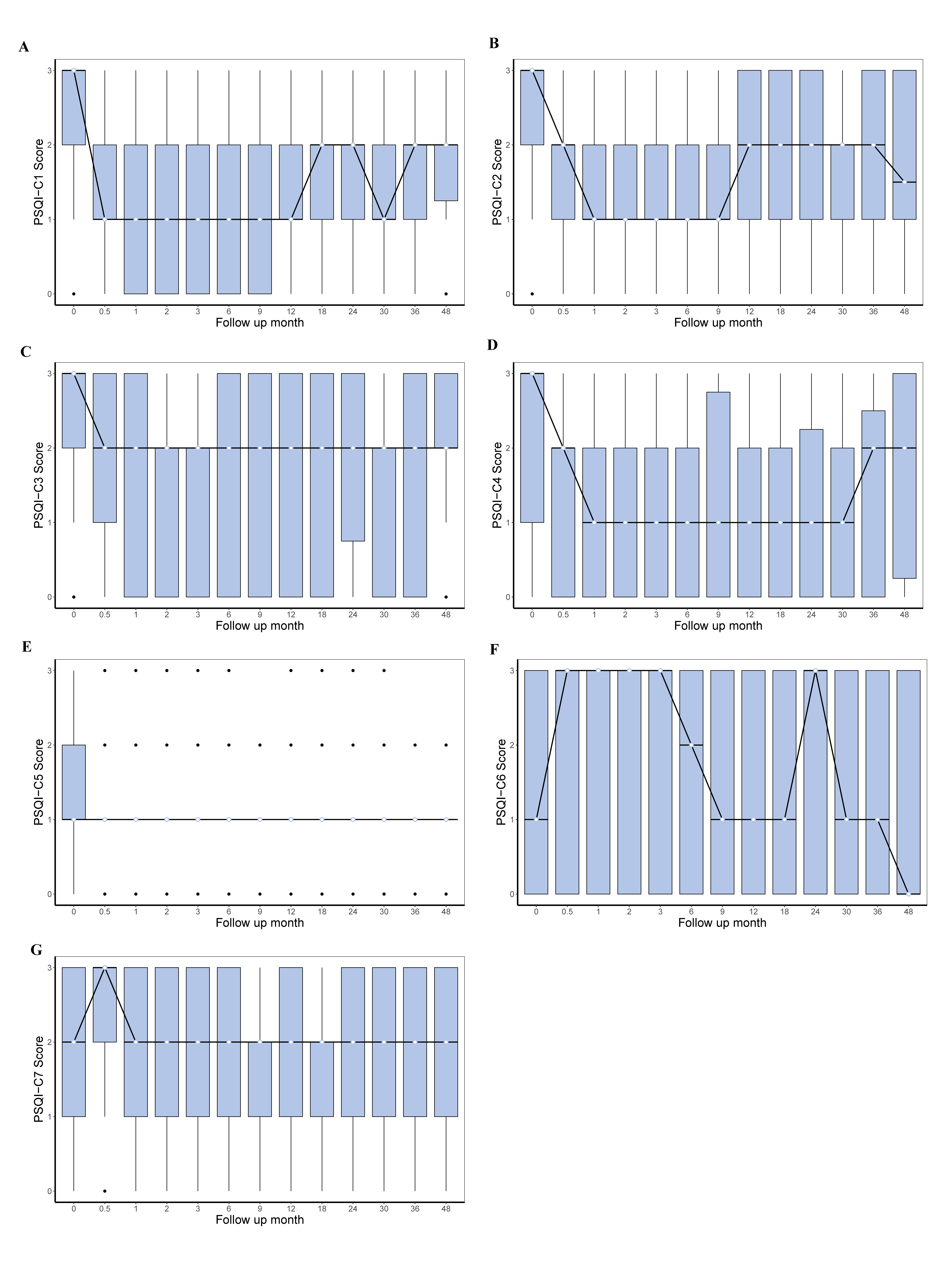

Supplement: Supplementary file 2 [file Image_1.tif]

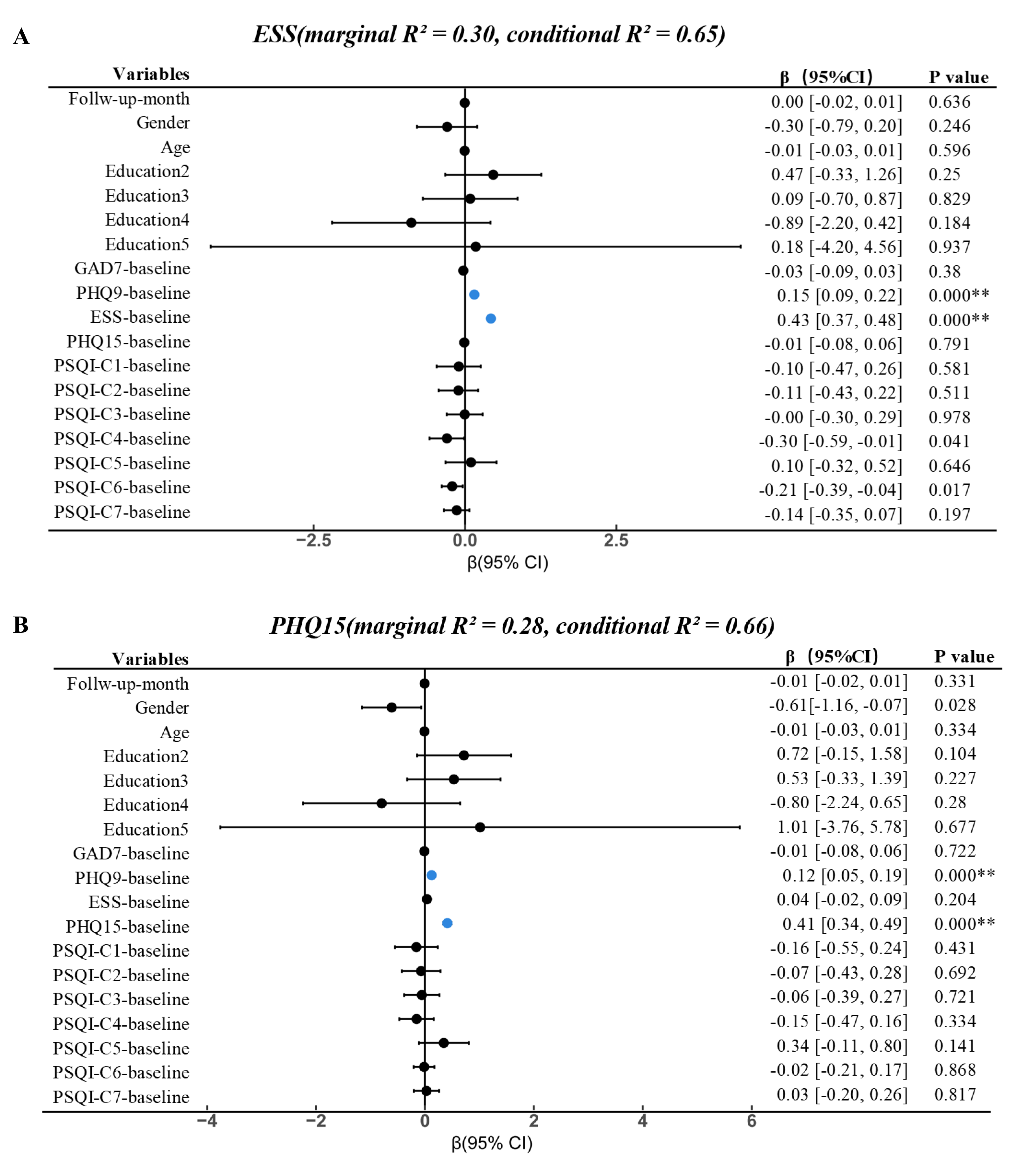

Supplement: Supplementary file 3 [file Image_2.tif]
